# Crystal Structure of Human Importin-α1 (Rch1), Revealing a Potential Autoinhibition Mode Involving Homodimerization

**DOI:** 10.1371/journal.pone.0115995

**Published:** 2015-02-06

**Authors:** Hideyuki Miyatake, Akira Sanjoh, Satoru Unzai, Go Matsuda, Yuko Tatsumi, Yoichi Miyamoto, Naoshi Dohmae, Yoko Aida

**Affiliations:** 1 Global Research Cluster Collaboration Promotion Unit, RIKEN, 2–1, Hirosawa, Wako-shi, Saitama 351–0198, Japan; 2 Protein Wave Corporation, 1–16–5 Nishitomigaoka, Nara 631–0006, Japan; 3 Graduate School of Medical Life Science, Yokohama City University, 1–7–29 Suehiro-cho, Tsurumi-ku, Yokohama 230–0045, Japan; 4 Viral Infectious Diseases Unit, RIKEN, 2–1, Hirosawa, Wako-shi 351–0198, Japan; 5 Department of Biochemistry and Molecular Biology, School of Biomedical Sciences, Monash University, Building 76 Level 2, Wellington Road, Clayton VIC 3800, Australia; 6 Laboratory of Nuclear Transport Dynamics, National Institute of Biomedical Innovation, 7–6–8 Saito-Asagi, Ibaraki-shi, Osaka 567–0085, Japan; Università degli Studi di Milano, ITALY

## Abstract

In this study, we determined the crystal structure of N-terminal importin-β-binding domain (IBB)-truncated human importin-α1 (ΔIBB-h-importin-α1) at 2.63 Å resolution. The crystal structure of ΔIBB-h-importin-α1 reveals a novel closed homodimer. The homodimer exists in an autoinhibited state in which both the major and minor nuclear localization signal (NLS) binding sites are completely buried in the homodimerization interface, an arrangement that restricts NLS binding. Analytical ultracentrifugation studies revealed that ΔIBB-h-importin-α1 is in equilibrium between monomers and dimers and that NLS peptides shifted the equilibrium toward the monomer side. This finding suggests that the NLS binding sites are also involved in the dimer interface in solution. These results show that when the IBB domain dissociates from the internal NLS binding sites, e.g., by binding to importin-β, homodimerization possibly occurs as an autoinhibition state.

## Introduction

In eukaryotic cells, the cytoplasm and the nucleus are divided by the nuclear membrane. The nuclear pore complex (NPC), situated on the nuclear membrane, acts as a gate that allows free diffusion of particles, such as small molecules and ions of less than ~9 nm [1–3]. Particles that are larger than 40 kDa and comprise proteins or protein–nucleic acid complexes are transported through the NPC by a variety of active transport mechanisms [4], [5]. Systems consisting of importin α/β have been intensively studied [6–8]. Human importin-αs (h-importin-αs) comprise at least seven isoforms: h-importin-α1 (Rch1/KPNA2), h-importin-α3 (Qip1/KPNA4), h-importin-α4 (KPNA3), h-importin-α5 (NPI-1/KPNA1), h-importin-α6 (KPNA5), h-importin-α7 (KPNA6), and h-importin-α8 (KPNA7) [9], [10]. In addition, at least 23 kinds of human importin-βs have been identified. In a typical scenario of importin-α/β transportation, the flexible N-terminus importin-β binding domain (IBB) binds to the NLS binding sites of importin-α in an autoinhibition manner. When the importin-α complexes to the importin-β with the IBB domain, it dissociates from the NLS binding sites. Then the cargo proteins tagged with the basic nuclear localization signal (NLS) sequence can bind to the exposed NLS binding sites on the surface of importin-α. Finally, hetero-trimer, importin-αβ and the cargo protein is formed.

Importin-α works as an adaptor molecule to connect importin-β and the NLS cargo, which makes it possible for the limited variety of importin-βs to bind to a large variety of NLS cargoes. The hetero-trimer is then transported into the nucleus through the NPC, with the importin-β working as an active engine. Such a transportation system ensures that larger cargoes (Mw > 40 kDa) can travel through the NPC into the nucleus [9–12]. There seems to be some mechanism in the isoforms of human importin-αs that is responsible for the diversity of binding to a variety of NLS cargoes, even though the whole structures of the subfamilies closely resemble each other. On the other hand, some importin-αs are known to form homodimers. The crystal structure of yeast importin-α was first reported as a homodimer [13]. In the homodimer, major and minor NLS binding sites are still exposed to solution; thus, NLS peptides can bind to the sites. In addition, *Xenopus* importin-α seems to form homodimers or multimers during purification [14]. However, the functional aspects of these multimerization properties of importin-αs have not been resolved.

Here, we report homodimeric human importin-α1 (h-importin-α1) by the use of X-ray crystallography and shed light on the solution state of the homodimerization in relation to NLS binding by using analytical ultracentrifugation (AUC) and isothermal titration calorimetry (ITC).

## Materials and Methods

### Expression system construction

Full-length human importin-α1 (h-importin-α1) (1–526) was generated from pGEX6P3/importin-α1-Flag, as previously described [15], by site-directed mutagenesis, resulting in a Phe-to-Stop substitution at amino acid 527. IBB domain-truncated h-importin-α1 (Δ-h-importin-α1) (1–10, 55–529) was generated from pGEX6P3/importin-α1-His_6_, as previously described, by inverse polymerase chain reaction (PCR) amplification using the following primers: forward, 5′*-TCA TTT CCT GAT GAT GCT ACT TCT CCG CTG-*3′, and reverse, 5′*-TGG TGT ATT AGC ATT CTC GTT GGT GGA CAT-*3′. The PCR product was digested with *Bam*HI and *Not*I and cloned into pGEX6P3. All constructs were verified by DNA sequencing.

### Protein preparation

h-importin-α1 (1–526) and ΔIBB-h-importin-α1 were overexpressed by *E*. *coli* strain BL21 (DE3) Codonplus-RIL (Stratagene) and purified as follows: Frozen cells (10 g) obtained from a 1 L culture were suspended in 100 mL of sonication buffer (50 mM tris(hydroxymethyl)aminomethane (Tris)-HCl, 500 mM NaCl, 1 mM dithiothreitol (DTT), 1 mM ethylenediaminetetraacetic acid (EDTA), pH 8.0) and lysed by sonication using an Ultrasonic Processor VCX 500 (Sonics & Materials, Inc.). The lysate was collected and centrifuged at 15,000 rpm for 1 h. The supernatant was filtered through a 0.45 μm HT Tuffryn membrane syringe filter (Pall Corporation). A GSTrap FF column (GE Healthcare) packed with Glutathione Sepharose 4 Fast Flow (GE Healthcare) was pre-equilibrated with phosphate-buffered saline (PBS) buffer. This lysate was then loaded onto a column at a flow rate of 1 mL/min. After washing with 50 mL wash buffer (50 mM Tris-HCl, 150 mM NaCl, 1 mM DTT, 10% glycerol, 1 mM EDTA, pH 8.0), bound GST-tagged h-importin-α1 was eluted with 30 mL elution buffer (50 mM Tris-HCl, 150 mM NaCl, 1 mM DTT, 10% glycerol, 1 mM EDTA, 5 mM reduced glutathione, pH 8.2). Elution fractions containing GST-h-importin-α1 and GST-ΔIBB-h-importin-α1 were pooled. The PreScission protease (GE Healthcare) was incubated with the pooled fractions according to the manufacturer’s instructions, and the solution was dialyzed against 2 L of digestion buffer (50 mM Tris-HCl, 150 mM NaCl, 1 mM DTT, 1 mM EDTA, 0.01% Triton X-100, pH 7.5) for 12 h at 277 K. The digested protein was filtered through a 0.45 μm HT Tuffryn membrane syringe filter and loaded onto a GSTrap FF column (GE Healthcare). The cleaved h-importin-α1 and ΔIBB-h-importin-α1 were collected in the flow-through and washed fractions. The protein samples were diluted to obtain an NaCl concentration of 75 mM and a pH of 8.0 with 20 mM Tris-HCl, 1 mM DTT, 1 mM EDTA, and 10% glycerol. The samples were loaded onto a Resource-Q column (GE Healthcare) packed with SOURCE 15Q (GE Healthcare) (S1 Fig.). For elution, the column was developed with a linear gradient from 0 to 500 mM NaCl in 20 mM Tris-HCl, 1 mM DTT, 1 mM EDTA, 10% glycerol, pH 8.0, at a flow rate of 0.2 mL/min. The eluted fractions of interest were pooled and concentrated with a Centriprep YM-10 centrifugal device (Amicon/Millipore) to a concentration of 10 mg/mL and used for the ITC and AUC-SV experiments. For the crystallization experiments, these fractions were filtered and loaded in a pre-equilibrated HiLoad 16/60 Superdex 75 size exclusion column (GE Healthcare) with 20 mM Tris-HCl, pH 8.0, 200 mM NaCl, and 5 mM DTT. The eluted fractions were collected and concentrated with the Centriprep YM-10 to 10 mg/mL.

### Peptide synthesis

SV40 NLS (PKKKRKV) and nucleoplasmin NLS (KRPAATKKAGQAKKKK) peptides were synthesized by stepwise solid-phase synthesis on a 433A peptide synthesizer (Applied Biosystems) using a standard 9-fluorenylmethyloxycarbonyl strategy (amino acid activated and coupled with 2-(1H-benzotriazol-1-yl)-1,1,3,3-tetramethyluronium hexafluorophosphate/ 1-hydroxybenzotriazole/N,N-diisopropylethylamine) on a 0.1 mM scale.

### Analytical ultracentrifugation (AUC)

AUC sedimentation velocity (AUC-SV) experiments were carried out in AUC buffer (0.1 M Tris-HCl, pH 8.0, 0.2 M NaCl, 1 mM tris(2-carboxyethyl)phosphine (TCEP)), using an Optima XL-I analytical ultracentrifuge equipped with two optical systems, the Rayleigh interference and absorbance systems (Beckman Coulter). For AUC-SV measurements, 10 mg/mL h-importin-α1 and ΔIBB-h-importin-α1 samples were diluted with the AUC buffer. Then, 4 mM SV40 and nucleoplasmin NLS solutions, dissolved in the AUC buffer, were added to the sample solution. The AUC-SV measurements were conducted at 50,000 rpm at a temperature of 293 K, using an An-50 Ti rotor featuring cells with a standard 12-mm charcoal-epon double sector centerpiece and sapphire windows. During the runs, changes in the concentration gradient were monitored with a Rayleigh interference optical system or absorbance at 280 nm. All of the AUC-SV raw data were analyzed by the program SEDFIT14.1, with the continuous *C*(*s*) distribution model [16]. The SEPHAT 10.58d program was used for analysis of the isotherm of weight-average s-values. A monomer-dimer self-association model was applied [17].

### Isothermal titration calorimetry (ITC)

We investigated the binding of SV40 NLS and nucleoplasmin NLS to h-importin-α1 and ΔIBB-h-importin-α1. ITC measurements were carried out using a MicroCal iTC_200_ (GE Healthcare) in 0.1 M Tris-HCl, pH 8.0, 0.2 M NaCl, and 5 μM DTT at 293 K. The cell chamber was loaded with 200 μL of 15 μM h-importin-α1 and titrated 20 times with 2 mM of SV40 NLS solution or 2 mM nucleoplasmin NLS solution, while stirring at 1,000 rpm. The data analysis was performed with Origin software equipped with the ITC (Fig. 3A). ΔIBB-h-importin-α1 (17 μM) was used for the ITC experiment with 40 titrations for SV40 NLS binding and 30 titrations for nucleoplasmin NLS (Fig. 3B, C).

### Crystallization of ΔIBB-h-importin-α1

The crystals of ΔIBB-importin-α1 were prepared at 293 K using the sitting drop vapor diffusion method with 5.0 μL of the concentrated protein solutions (10 mg/mL) and an equal volume of precipitant buffer (50 mM 2-(N-morpholino)ethanesulfonic acid (MES) pH 5.5, 100 mM ammonium sulfate, 10 mM MgCl_2_, 15–20% (w/v) polyethylene glycol (PEG) 8000). Crystals appeared within 2 days and grew to a size of 0.5 mm in 2 weeks.

### Diffraction data collection, structure determination and validation

All diffraction data were collected at the BL26B2 station of SPring-8 using the mail-in data collection system [18], [19]. The crystals were soaked in the solution containing 30% (w/v) PEG 8000 as a cryoprotectant and flash-frozen at 100 K. The diffraction data were processed using the HKL2000 package [20]. The molecular replacement technique was executed using the MrBump package [21] using the truncated molecular model (84–476) of importin-α5 (PDB ID: 2JDQ) as a template, which gave two solutions in the asymmetric unit. The initial model was improved using RESOLVE [22] and further refined using LAFIRE [23] and ARP/wARP packages [24]. The model was manually rebuilt with the help of XtalView [25] and Coot [26], and refined with CNS [27] and PHENIX [28]. The current model of the ΔIBB-importin-α1 involves the residues of 75–496 (chain A) and 75–497 (chain B) with an *R/R*_free_ factor of 0.193/0.221. The structural validation was performed with the Procheck [29] and MolProbity [30] programs. The coordinate and structure factors of ΔIBB-h-importin-α1 have been deposited at the Protein Data Bank with PDB ID: 3WPT.

## Results

### Expression and purification of h-importin-α1 and ΔIBB-h-importin-α1

h-importin-α1 and ΔIBB-h-importin-α1 were expressed by the *E*. *coli* strain BL21 (DE3) Codonplus-RIL (Stratagene). S1 Fig. shows the chromatograms corresponding to elution from an anion exchange column (Resource Q) used for the purification of h-importin-α1 and ΔIBB-h-importin-α1.

### AUC-SV experiments

**Fig 1:**
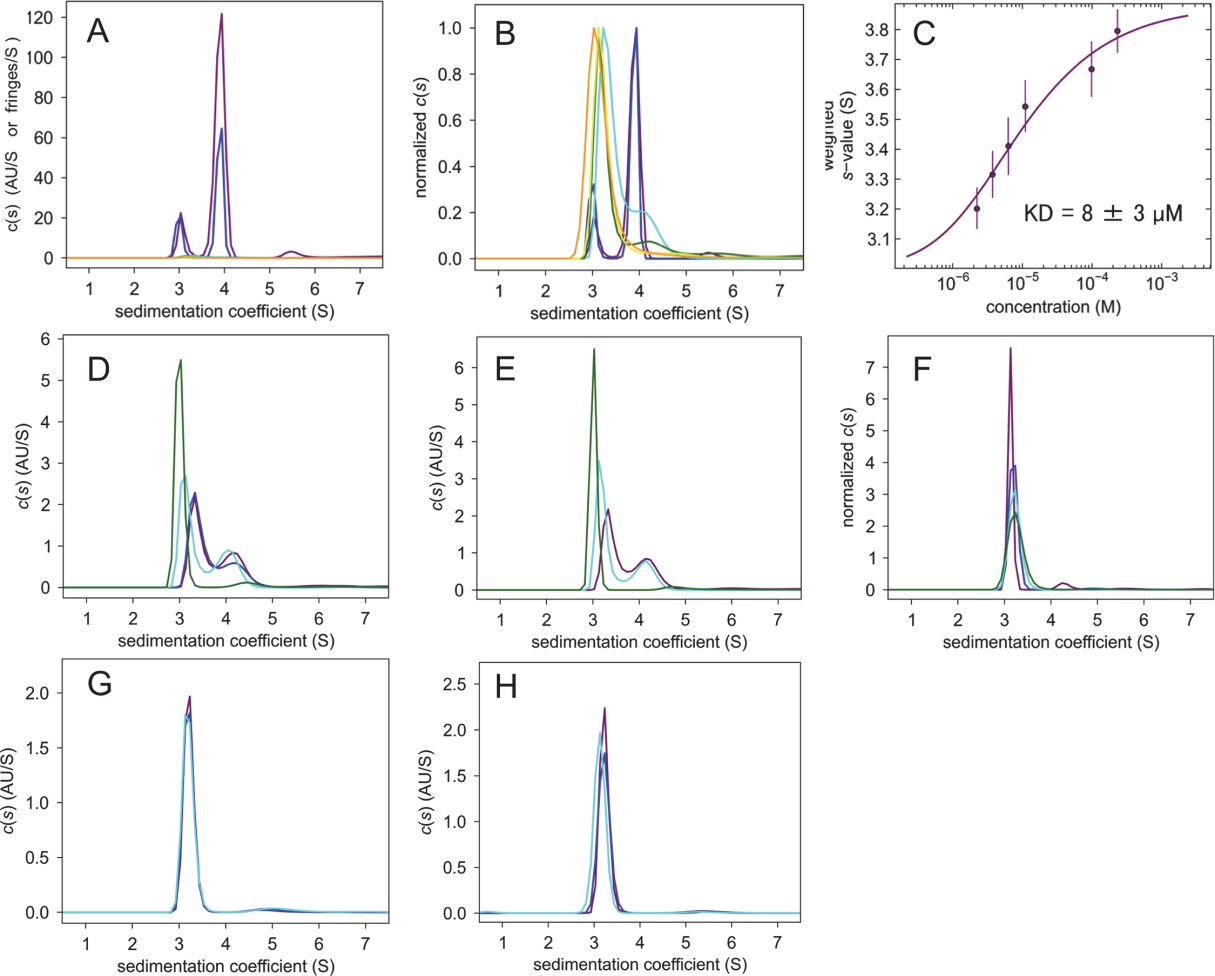
AUC-SV measurements. (A) c(s) distributions of ΔIBB-h-importin-α1, 247 μM (purple), 100 μM (blue), 14 μM (cyan), 11 μM (green), 7.3 μM (yellow) and 4.2 μM (orange). (B) Normalized c(s) distributions of ΔIBB-h-importin-α1. (C) KD value estimation by the fitting curve of the sw vs protein concentration. (D) 25 μM ΔIBB-h-importin-α1 + SV40 NLS of 0 μM (purple), 1 μM (blue), 10 μM (cyan) and 100 μM (green). (E) 25 μM ΔIBB-h-importin-α1 + nucleoplasmin NLS of 0 μM (purple), 10 μM (cyan), and 100 μM (green). (F) Normalized c(s) distributions of h-importin-α1, 61 μM (purple), 34 μM (blue), 8.6 μM (cyan), and 4.3 μM (green). (G) 10 μM h-importin-α1 + SV40 NLS of 0 μM (purple), 10 μM (blue), 100 μM (cyan). (H) 10 μM h-importin-α1 + nucleoplasmin NLS of 0 μM (purple), 10 μM (blue), 100 μM (cyan).

**ΔIBB-h-importin-α1.** The AUC-SV for ΔIBB-h-importin-α1 shows that it is in a concentration-dependent monomer–dimer equilibrium (Fig. 1A, B). The *C*(*s*) distributions are characterized by two peaks, one at 3.1–3.3 S and one at 3.9 S; the relative sizes of the peaks vary with protein concentration, and they are interpreted as corresponding to monomer and dimer, respectively. The *C*(*s*) distributions were used to create a weight average S value (sw) isotherm. We calculated the sw by integration of the *C*(*s*) curves between 2.5 S and 5.0 S for each protein concentration, and then plotted the sw vs protein concentration using SEDPHAT (Fig. 1C). The monomer–dimer self-association model was applied for the curve fitting. The monomer–dimer KD is estimated to be 8 ± 3 μM. The NLS ligands, SV40 and NP, shift the equilibrium to the monomer side (Fig. 1D, E). These results show that the NLS binding sites are involved in the dimer interface in solution.

**h-importin-α1.** However, h-importin-α1 remains monomeric in a variety of protein concentration (Fig. 1F). Neither of the SV40 nor NP NLS peptides changed the monomeric state (Fig. 1G, H). These results show that the self-bound IBB domain autoinhibits not only NLS binding but also homodimerization.

### X-ray crystallography of ΔIBB-h-importin-α1

**Fig 2:**
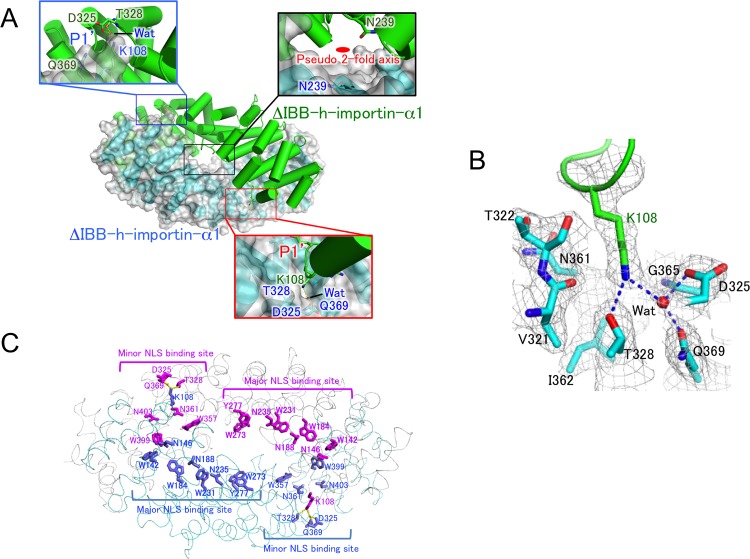
Crystal structure of homodimeric ΔIBB-h-importin-α1. (A) The closed homodimer structure of ΔIBB-h-importin-α1. One of the protomers is shown as a surface drawing. Both of the P1′-binding pockets are depicted in close-up views. The K108 inserts in the P1′-binding pocket, making hydrogen bonds with D325, T328, and Q369 of another protomer, and Wat (a water molecule). The pseudo 2-fold axis is drawn in the close-up view for the center of the dimer. (B) The P1′ binding pocket with 2fo-fc electron density maps (>1.0σ). Residues involved in the P1′-binding pocket are V321, T322, D325, T328, N361, I362, and Q369. One water molecule is depicted as Wat. The K108 of another protomer is inserted into the P1′-binding pocket, making hydrogen bonds depicted with dotted blue lines. (C) A ribbon drawing of the homodimeric ΔIBB-importin-α1 in cyan and gray. Residues of each protomer are in blue and orange. Major and minor NLS binding sites are indicated. Residues involved in the major NLS binding sites are W142, N146, W184, N188, W231, N235, W273, and Y277 in bold characters. The residues in the minor NLS biding sites are D325, T328, W357, N361, Q369, W399, and N403. Each of the K108 makes hydrogen bonds with D325, T328, Q369, and one water molecule in the minor NLS binding site of another protomer. Because the major and minor NLS binding sites are extensively buried in the dimerization interface as shown in the drawing, NLS signals are inaccessible to the sites. All molecular pictures were prepared with PyMol [40].

**Fig 3:**
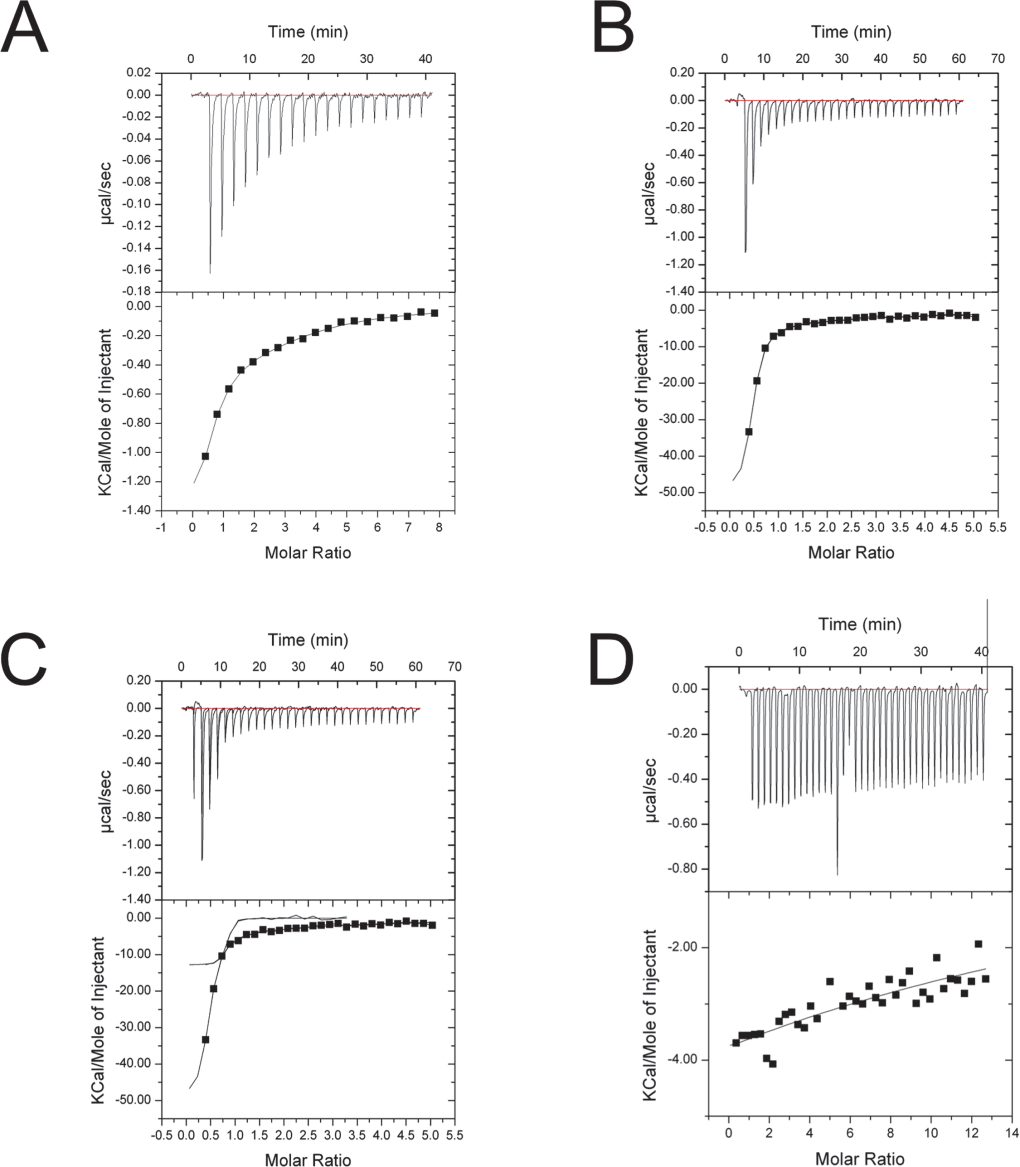
ITC fitting curves. (A) h-importin-α1 + SV40 NLS peptide. (B) ΔIBB-h-importin-α1 + SV40 NLS peptide. (C) ΔIBB-h-importin-α1 + nucleoplasmin NLS. (D) h-importin-α1 + nucleoplasmin.

Only the diffractive crystals of ΔIBB-h-importin-α1 were obtained, not those of h-importin-α1. The crystals diffracted X-rays to 2.6 Å resolution in SPring-8. The molecular replacement technique was used to solve the structure (Table 1). The crystal structure of ΔIBB-importin-α1 is shown in Fig. 2A. The pseudo 2-fold axis lies in close proximity to the N239 of chains A and B, which are in the disallowed region in the Ramachandran plot. Structural stress is probably put on them owing to the dimerization. The K108 residue is extensively involved in the dimer formation and is represented in the close-up views. The side chain of K108 enters the P1′ site of the minor NLS binding site [31], making hydrogen bonds with D325, T328, and Q369 of another protomer and one water molecule (Fig. 2B). The P1′ site comprises V321, T322, D325, T328, N361, I362, G365, and Q369, which are conserved in the minor NLS binding site of importin-α proteins (Fig. 3). The major (W142, N146, W184, N188, W231, N235, W273, Y277) and minor (W357, N361, W399, N403) NLS binding sites are buried in the dimer interface (Fig. 2C), thus NLS peptides cannot access the NLS binding sites. Because the major NLS binding site involves typically P1-P7 binding pockets [32], it has higher NLS binding affinity than the minor NLS binding site with P1′-P2′.

**Table 1:** Crystallographic data collection and refinement statistics of ΔIBB-h-importin-α1.

Space group	Tetragonal *P*4_3_2_1_2
Cell constants (Å)	*a = b* = 139.30, *c* = 141.29
Wavelength (Å)	1.00
Resolution (Å)	50.0–2.60 (2.69–2.60)
Oscillation angle (deg.)	1.0
*σ*cut-off	0.0
*R*_merge_ ^a^	0.065 (0.391)
No. of measurements	352,972 (3,701)
No. of independent reflections	40,652
Completeness (%)	96.96 (90.25)
Multiplicity	8.7 (4.8)
Mean <I/ σ(I)>	31.83 (4.86)
Wilson B-factor (Å)	48.59
Refinement statistics	
Resolution (Å)	46.7–2.63 (2.79–2.63)
No. of reflections used	40,616 (5,982)
Completeness (%)	96.96 (90.25)
*R*_work_ ^b^	0.1930 (0.2843)
*R*_free_ ^c^	0.2207 (0.3062)
No. of atoms	
Protein (chain A, chain B)	6,496, 6,507
PEG	11
Water	192
Average B factor	
Protein (chin A, chain B)	45.879, 38.836
PEG	71.622
Water	39.057
R.m.s. deviations	
Bond lengths (Å)	0.003
Bond angles (°)	0.671
Ramachandran plot (%)	
Favored region	95.6
Allowed region	4.04
Outer region	0.36
Clashscore^d^	12.08

^a^ *R*_merge_ = ΣΣ_*i*_ ||*I*(*h*)—*I*(*h*)_*i*_ | / ΣΣ_*i*_ *I*(*h*), where *I*(*h*) is the mean intensity after rejections.

^b^ *R*_work_ = Σ| F_p_—F_pc_ /Σ |F_p_|.

^c^ *R*_free_, the same as *R*_work_ but calculated on 4.93% of data excluded from refinement.

^d^ Clashscore, calculated by MolProbity.

Values in parentheses are for highest resolution shells.

**Fig 4:**
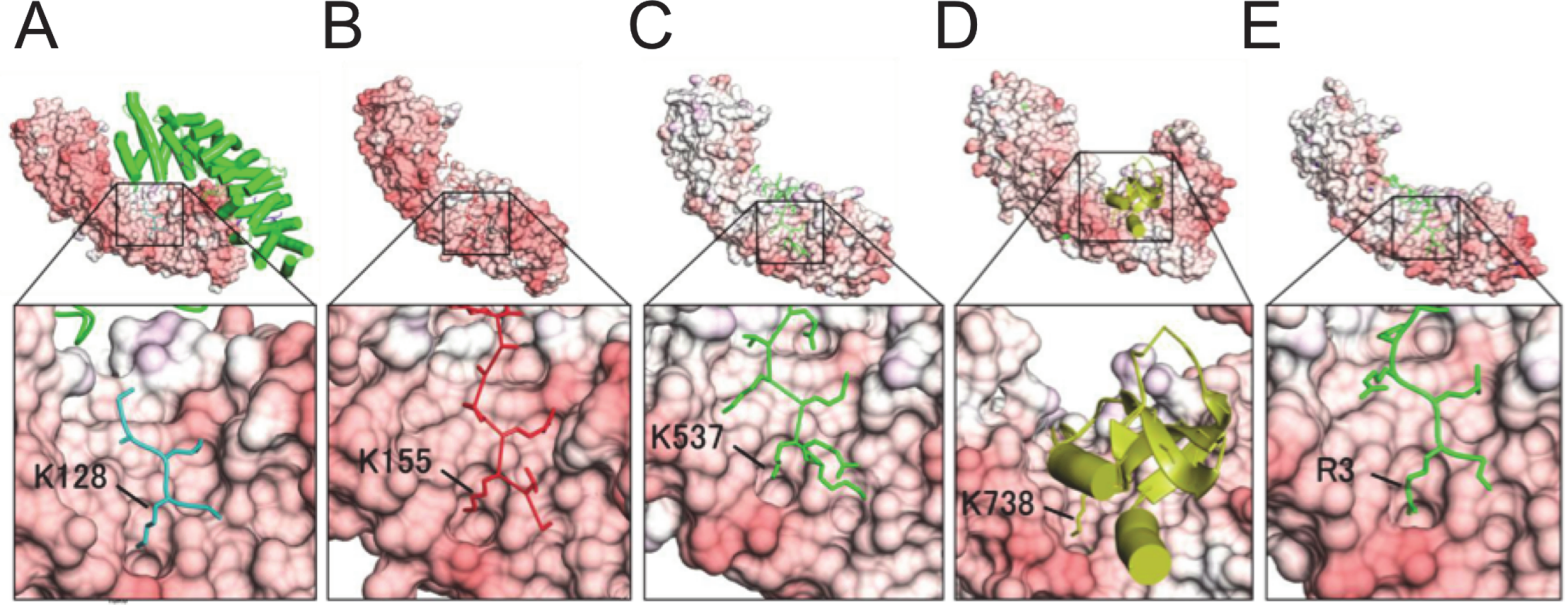
Crystal structures of complexes of importin-αs and NLS ligands with close-up views showing the P1′-binding pocket in the minor NLS binding sites. (A) K128 of SV40 NLS peptide (Yeast importin-α: PDB ID: 1BK6), (B) K155 of *Xenopus* nucleoplasmin NLS (Yeast importin-α: 1EE5), (C) K537 of *Xenopus* nucleoplasmin NLS (importin-α2: 1PJN), (D) K738 of influenza virus polymerase subunit Pb2 (importin-α5: 2JDQ), and (E) R3 of the IBB domain of the CBP20 (ΔIBB-importin-α1: 3FEY).

The P1′-binding pocket in the minor NLS binding site plays a common role in a variety of importin-αs; the P1′ accepts the K/R residues of the typical NLS signal motif KRXX [31–33], (Fig. 4). In the present crystal structure of ΔIBB-h-importin-α1, each of the K108 residues enters into the P1′-binding pocket of another protomer so that the minor NLS binding sites are in a state of autoinhibition (Fig. 2B), and, together with the major NLS binding sites, are buried in the dimer interface (Fig. 2C). As a result, the closed homodimer is stabilized owing to the limited access of NLS peptides to the major and minor NLS binding sites.

### Isothermal titration calorimetry (ITC) assay for binding of NLS peptides to h-importin-α1 and ΔIBB-h-importin-α1

**h-importin-α1.** We carried out a binding assay of h-importin-α1 and ΔIBB-h-importin-α1 to study autoinhibition resulting from homodimerization. SV40 NLS bound to h-importin-α1 with a KD value of ~100 μM (Table 2). While the fitting to the ITC data, one-site binding model was used because two-site model did not converge to adequate values. The AUC-SV experiments reveal that h-importin-α1 is monomeric during SV40 NLS binding (Fig. 1G). This result suggests that both the IBB domain and the NLS peptides bind to the internal NLS binding sites and prevent homodimerization.

**Table 2:** Binding parameters of h-importin-α1 and ΔIBB-h-importin-α1 by ITC.

Ligand: SV40 (PKKKRKV)			
Protein	KD (μM)	N	ΔH (kcal/mol)
h-importin-α1	102.1±11.0	0.8±0.2	-3.2± 0.9
ΔIBB-h-imporin-α1	5.5±2.0	0.5±0.1	-57.8±20.8
Ligand: Nucleoplasmin NLS (KRPAATKKAGQAKKKKK)			
Protein	KD (μM)	N	ΔH (kcal/mol)
h-importin-α1	N.D.		
ΔIBB-h-imporin-α1	0.14±0.05	0.8±0.0	-12.8±0.3

KD: dissociation constant,

N: number of binding sites,

ΔH: binding enthalpy,

N.D. not detectable.

On the other hand, the bipartite nucleoplasmin NLS peptide did not bind to h-importin-α1 (Fig. 3D). This result suggests that NLS binding is restricted by the IBB domain.

**ΔIBB-h-importin-α1.** ΔIBB-h-importin-α1 bound to SV40 NLS with KD = ~6 μM and to nucleoplasmin NLS peptides with KD = ~0.14 μM (Table 2). During the NLS binding, the monomer–dimer equilibrium shifted to the monomer side (Fig. 1D, E), which suggests that the NLS binding sites are involved in the dimer interface even in solution, as in the crystal structure.

## Discussion

### Homodimerization found in importin-αs

It has been shown or suggested that the importin-αs from yeast and *Xenopus* eggs form dimers. Yeast importin-α (PDB IB: 1BK6) provided the first crystal structure of an importin-α that was shown to dimerize not only in the crystal but also in solution, as revealed by dynamic light scattering (DLS) [13]. The SV40 NLS peptides are, however, able to bind to the NLS binding sites because the homodimer is an “open” structure where the NLS binding sites are exposed to the solution (Fig. 4A). The open homodimerization probably attributes to H119 at the top of the loop; this loop corresponds to the K108 loop of ΔIBB-importin-α1. H119 cannot enter the P1′-binding pocket in the minor NLS binding site, and this inability causes the protomers to slide along the long axis of the molecules. The NLS binding parameters of yeast importin-α were analyzed by fluorescence depolarization assay and it was found that yeast importin-α (1–542) binds to neither SV40 NLS-GFP nor Myc NLS-GFP [34]. On the other hand, yeast ΔIBB-importin-α (89–530) binds to both SV40 and Myc NLS, with KDs of 9 ± 4 nM and 6 ± 3 nM, respectively [35]. In addition, an enzyme-linked immunosorbent assay (ELISA)-based assay revealed that yeast importin-α (1–542) does not bind to SV40 NLS, whereas yeast ΔIBB-importin-α (88–530) binds very tightly [35]. These results suggest that the IBB domain is mainly responsible for the autoinhibition activity of yeast importin-α, while the “open homodimerization” does not restrict the NLS binding. Thus, the NLS binding affinity of yeast importin-α changes dramatically from 0% (off), when the IBB autoinhibits itself, to 100% (on), when it does not, that is keenly contrast to the closed-homodimer of h-importin-α1.

Moreover, it has been reported that wild importin-α from *Xenopus* eggs is homodimerized during purification [14], and a variety of studies have revealed that purified *Xenopus* importin-α is in dimer–monomer equilibration in the concentration range of 0.6~1.2 mM, with a dissociation constant KD of ~20 μM [36]. We found that the *Xenopus* importin-α has an arginine residue (R105) that is aligned to the K108 of importin-α1 (S2 Fig.). Thus, the R105 possibly interacts with the P1′-binding pocket of another protomer, promoting the closed-homodimerization.

The first structural knowledge about the autoinhibition mechanism was obtained from the crystal structure of mouse-importin-α2 (m-importin-α2) [37], [38]. In that crystal structure, the IBB domain binds to the major, but not the minor, NLS binding site; thus the NLS binding affinity is partly autoinhibited. It has been unclear whether m-importin-α2 forms a homodimer or not [37], although the m-importin-α2 has a K102 and the P1′-binding pocket consists of the same residues as in h-importin-α1 (S2 Fig.). Further studies should be addressed about the multimerization property of the m-importin-α2.

It is reported that ΔIBB-h-importin-α1 (70–529) complexes with the cap-binding complex (CBC) [39]. In the ΔIBB-h-importin-α1 (70–529)-CBC complex, the NH1 and NH2 of R3 (P1′) in the canonical type of NLS (RRXX; P1′P2′XX) at the N-terminus of CBC (CBP80 + CBP20) form hydrogen bonds with OD2 of D325 and OG1 of T328 in the ridge of the P1′-binding pocket (Fig. 4E), which may shift the monomer-dimer equilibration to the monomeric side.

### A possible autoinhibition state of h-importin-α1

**Fig 5:**
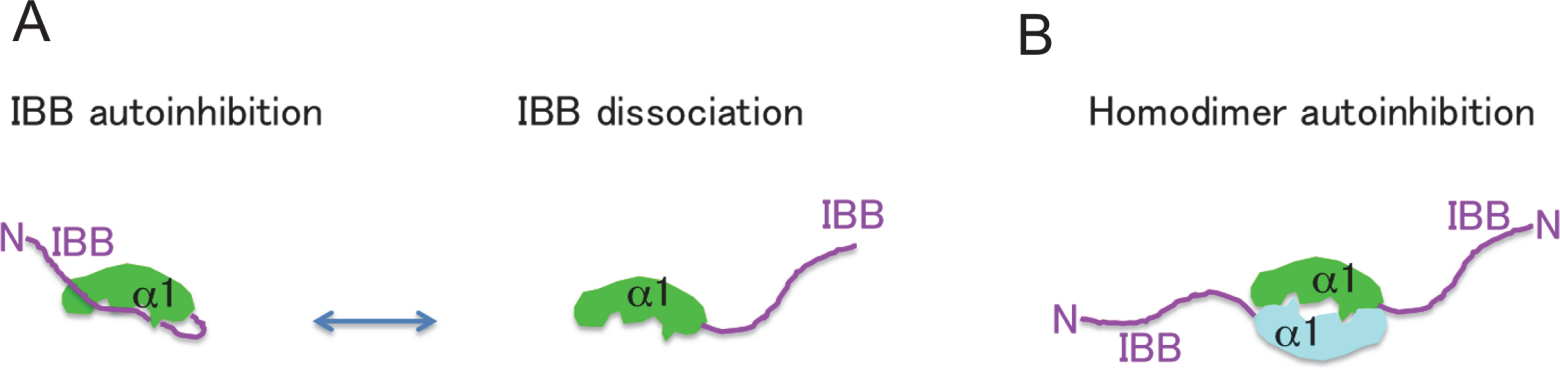
Autoinhibition states of h-importin-α1 by IBB binding and homodimerization. (A) A conventional scheme of autoinhibition by self-binding of IBB domain. The IBB domain bound in the NLS binding sites autoinhibits NLS binding. In addition, the current work reveals the IBB domain also prevents it from homodimerization. In turn, the bound IBB domain dissociates from the NLS binding sites by binding of NLS peptides and/or importin-β. (B) A potential homodimer autoinhibition mode. The current study reveals that a novel autoinhibition state by homodimerization, which possibly corresponds to NLS binding regulation.

Together with the results obtained from the AUC-SV, ITC, and crystallography, we can add a potential autoinhibition mode of the h-importin-α1 to the conventional scenario, as shown in Fig. 5. In the canonical scheme, the self-bound IBB domain autoinhibits NLS binding to the NLS binding sites in h-importin-α1. Our current study reveals that the self-bound IBB domain also autoinhibits homodimerization. The IBB domain is dissociated from the NLS binding sites, *e*.*g*., by the competitive binding of NLS peptides to the NLS binding sites and/or the binding of the IBB domain to importin-β (Fig. 5A). Upon the IBB domain dissociation, the h-importin-α1 can homodimerize as an autoinhibition state (Fig. 5B), which possibly lowers the KD (~6 μM) for NLS comparing to that of yeast (~10 nM) [34]. Through the association/dissociation of the IBB domain and molecular h-importin-α1, the NLS binding to the h-importin-α1 is controlled by both of the self-binding of IBB domain and homodimerization.

## Conclusions

In h-importin-α1, NLS binding affinity is autoinhibited by the self-binding of IBB domain. In addition, the closed homodimerization possibly works as an autoinhibition state when the IBB domain dissociates from the NLS binding sites. There still remains unresolved subjects whether the current closed-homodimer is physiologically relevant *in vivo*. Further studies are required to address the functional aspects of the closed-homodimer, by mutagenesis and cellular biological experiments.

## Supporting Information

S1 Fig Anion exchange chromatography with the Resource Q column and fraction characterization by 12.5% sodium dodecyl sulfate polyacrylamide gel electrophoresis (SDS-PAGE). (A) h-importin-α1. Each number assigned to the resulting elution position coincides with the lane of 12.5% SDS-PAGE. The blue line depicts the affinity column chromatography elution profile and the green line depicts the gradient of the NaCl concentration of the elution buffer. Fractions 39–44 were merged and concentrated. (B) ΔIBB-h-importin-α1. Fractions 40–45 were merged and concentrated.
(EPS)

S2 Fig Sequence alignment of importin-αs, with residue numbers and secondary structures of h-importin-α1. Sequences of human importin-α1 (UniProtKB: P52292), mouse importin-α2 (P52293), yeast importin-α (Q02821), and *Xenopus* importin-α1 (P52171). The position of K108 of importin-α1 is represented by a blue triangle. The residues (V321, T322, D325, N361, I362, G365) involved in the P1′-binding pocket around the minor NLS binding site are conserved among the importin-αs. Thus, the P1′-binding pocket should be conserved.
(EPS)
